# Texture-Induced Corrosion Resistance of Dissimilar AA7204/AA6082 Friction Stir Welded Joints

**DOI:** 10.3390/ma16186183

**Published:** 2023-09-14

**Authors:** Liqun Guan, Manfa Yuan, Jin Zhang, Yunlai Deng, Xuehong Xu, Li Wan

**Affiliations:** 1 Institute of Intelligent Manufacturing, Suzhou Jianxiong Vocational and Technical College, Suzhou 215400, China; shangguanliqun@163.com; 2 Guangdong Fenglu Aluminium Co., Ltd., Foshan 528133, China; 3 Enterprises Key Laboratory of Guangdong Aluminum Profile Processing and Equipment, Foshan Sanshui Fenglu Aluminium Co., Ltd., Foshan 528133, China; 4 Light Alloy Research Institute, Central South University, Changsha 410083, China; 5 School of Materials Science and Engineering, Central South University, Changsha 410083, China

**Keywords:** friction stir welding, in situ corrosion test, texture configuration, corrosion behavior

## Abstract

The quasi in situ EBSD test was applied to study the effect of grain orientation on corrosion behaviors of the thermomechanically affected zone I (TMAZ I) of dissimilar AA6082/AA7204 friction stir welding (FSW) joints in this work. The results show that the structure with grain orientation close to the brass texture ({110}<112>) has excellent corrosion resistance, which contributes to the better corrosion performance of the TMAZ I of the 7204-AS joint than the 7204-RS joint. Furthermore, the brass texture around by S texture ({213}<364>) in the TMAZ I of the 7204-AS joint is slightly corroded, and the orientation of the remaining structure is closer to the ({110}<112>) than before, which indicates that the corrosion, like deformation, is carried out alongside the {110} plane for the structure with grain orientation near {110}<112>. Those findings could provide new insight into the designed FSW joints and improve the corrosion resistance of the wrought aluminum alloy.

## 1. Introduction

Aluminum and aluminum alloys, which are lightweight, fuel efficient, and have good structural strength and low emissions, have a large demand in the aircraft and automotive industries [1,2]. Al-Mg-Si alloys are extensively used in structural materials for automobiles and railway vehicles due to their good corrosion resistance, excellent weldability, and ductility [3,4,5]. Al-Zn-Mg alloys are common structural materials used in high-speed railways, subways, and other vehicle transport due to their high specific strength, good toughness, and good weldability [6,7,8]. Therefore, welding Al-Mg-Si and Al-Zn-Mg alloys is inevitable in industrial production. However, welding Al-Mg-Si and Al-Zn-Mg alloys are difficult because of their different mechanical, physical, and chemical properties [9,10].

Friction stir welding (FSW) has the potential to weld Al-Mg-Si and Al-Zn-Mg alloys due to its low energy consumption, good weld qualities, environmental friendliness, and ease of use in multifunctional welding positions, which reduce the presence of distortions and residual stresses [7,11,12,13,14]. Due to the effect of the severe plastic deformation and thermal cycle, the typical welded joint of Al-Mg-Si and Al-Zn-Mg alloys includes a base metal (BM), a heat-affected zone (HAZ), a thermomechanically affected zone (TMAZ), and a nugget zone (NZ), and the HAZ and TMAZ will lead to the deterioration of the mechanical and corrosion resistance [15,16,17,18,19]. However, the corrosion behavior of Al-Mg-Si/Al-Zn-Mg joints has not been deeply studied, while studies have been carried out on the corrosion resistance of Al-Zn-Mg joints [17,18,20]. These studies show that the HAZ [17] and TMAZ [18] on the side of Al-Zn-Mg alloys have maximum corrosion susceptibility. The structure of the TMAZ is affected by the mechanical stirring of the stirring needle and the welding thermal cycle during the welding process. The TMAZ can be divided into two parts: TMAZ I, which is near the HAZ, and TMAZ II, which is near the NZ. The HAZ is subjected to a thermal cycle and results in slight grain growth, which will lead to the weakening of corrosion performance [17,18,21,22]. TMAZ I is subjected to a higher temperature thermal cycle and shear deformation, and the grain structure is characterized by fiber torsion, which will lead to the further deterioration of corrosion performance. Therefore, the study of the corrosion behavior of TMAZ I is of great theoretical and engineering significance.

Furthermore, FSW is a welding method with unsymmetrical thermal and mechanical cycles, so the positions of BMs on the AS (Advancing Side) or RS (Retreating Side) will influence the inhomogeneous microstructures and material flow across the welded joint due to their different thermophysical properties (such as specific heat and thermal conductivity) and mechanical properties (such as deformation ability, strength/hardness, and ductility) [7,15,23]. In particular, the grain orientation of the NZ and TMAZ [22,24,25] is greatly affected by the placement position of the BM. There are two main types of texture in FCC metal: (i) the plane-strain texture, including the brass ({011}<211>), copper ({112}<111>), and S ({123} <634>) textures, and (ii) the recrystallization texture, including cube ({001}<100>) and goss ({110}<001>) [26,27,28,29,30,31]. Al alloy corrosion usually involves precipitates, constituent particles [4,32,33], and texture [34,35,36]. Refs. [34,35] found that the effect of texture on corrosion property depends on the surface energy. Additionally, residual stress is generally retained near the welded joints [37,38], and it would be very interesting to study whether the residual stress will affect the corrosion resistance. The effect of grain orientation on the corrosion behavior of magnesium alloys [39,40,41,42] and steel [43,44,45,46] has been deeply studied, while the effect of grain orientation on the corrosion resistance of aluminum alloys [47] has rarely been reported. Therefore, it is meaningful to research the effect of the base metal configuration on the grain orientation and corrosion resistance of Al-Mg-Si/Al-Zn-Mg dissimilar friction stir welded joints.

In this study, FSW joints of AA7204-AS and AA7204-RS were successfully fabricated. To evaluate the corrosion resistance, a quasi in situ corrosion test, which was carried out by electron backscatter diffraction (EBSD), was used to study the uncorroded microstructures on the RD-ND surface of TMAZ I of the AA7204 side. Therefore, the texture-induced corrosion resistance of dissimilar AA7204/AA6082 friction stir welded joints was discussed in depth.

## 2. Experimental Procedures

### 2.1. Welding Process

As-received materials are AA6082 and AA7204 sheets, and their chemical composition is shown in Table 1. The AA6082 and AA72204 sheets were obtained with peak aging and natural aging after rolling, which were purchased from Northern Light Alloy Company.

**Figure 1:**
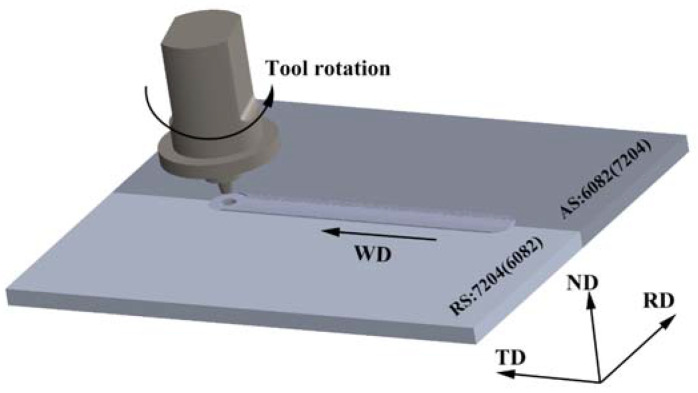
Schematic illustration of FSW.

The dimensions of the sheets were 350 mm × 150 mm × 12 mm, and all sheets were cleaned with alcohol to eliminate oil contamination before welding, which was beneficial for reducing the S-line defects in the NZ of FSW joints. A schematic illustration of FSW is shown in Figure 1. The two base metals were butt-joined using an FSW machine (FSW-HT-JM16 × 8/1, Aerospace Engineering Equipment Co., Ltd., Suzhou, China). The welding direction (WD) was perpendicular to the rolling direction (RD) of the sheet. The diameter of the shoulder was 26 mm, and the length of the pin was 12 mm. The diameters of the pin root and pin tip were 12 mm and 7.5 mm, respectively. A rotation rate of 450 rpm and a welding speed of 150 mm/min were conducted in the FSW process. Two types of joints, AA7204-RS and AA7204-AS, were welded.

After FSW, the macrostructures of the 7204-AS and 7204-RS joints were observed. First, the RD-ND surfaces were ground with sandpaper of grit size 80 to eliminate machining marks. Then, the sandpaper of grit sizes 320, 800, and 1500 was used to polish the sample surface in turn, and each grinding completely covered the previous grinding trace and was perpendicular to the previous grinding trace. Next, the welded joints were soaked in a 20% sodium hydroxide solution for approximately 10 min, and then the welded joints were soaked in a 10% nitric acid solution until the corrosion products on the surface were washed. The organizational streamline of the joints could be seen by the naked eye. Finally, the macrostructures of the 7204-AS and 7204-RS joints were obtained by optical microscopy (OM, OLYMPOS-DSX500, Tokyo, Japan), which was capable of automatically photographing and jigsaw puzzling.

### 2.2. Quasi In Situ Corrosive Tests

The RD-ND surfaces of the TMAZ I of the AA7204 side in the 7204-AS and 7204-RS joints were selected for the quasi in situ corrosion test, and were polished sequentially on 80 #, 320 #, 800 #, and 1500 # water abrasive paper and 800 # metallographic abrasive paper, and then mechanically polished with diamond grinding paste. Before the quasi in situ corrosion test, a ZEISS-EVOM10 scanning electron microscope (SEM, Oberkochen, Germany) was used to directly observe the morphology of the surface, and electron backscatter diffraction (EBSD) was performed. A sharp needle with a diameter of approximately 100 μm in the SEM was used to position the observation field. Next, the samples were electropolished at 20 V for 6 s in a solution of 10% perchloric acid and 90% ethanol to remove surface stress and were then immersed in a 3.5% NaCl solution at 25 °C for 0, 1, 2, and 4 h immediately to prevent corrosion in the air. Then, EBSD patterns were obtained by a ZEISS EVO M10 SEM with an OXFORD EBSD detector (SEM, Oberkochen, Germany) at the same position after each immersion. The vacuum environment inside SEM can ensure that corrosion does not occur during microstructure characterization, while corrosion occurs during immersion. The grain structure and texture were analyzed by HKL Channel 5 software (2015), and the grain structure with the orientation difference angle (ODA) between grain orientation and standard texture orientation within 10° was defined as the corresponding texture.

## 3. Results

**Figure 2:**
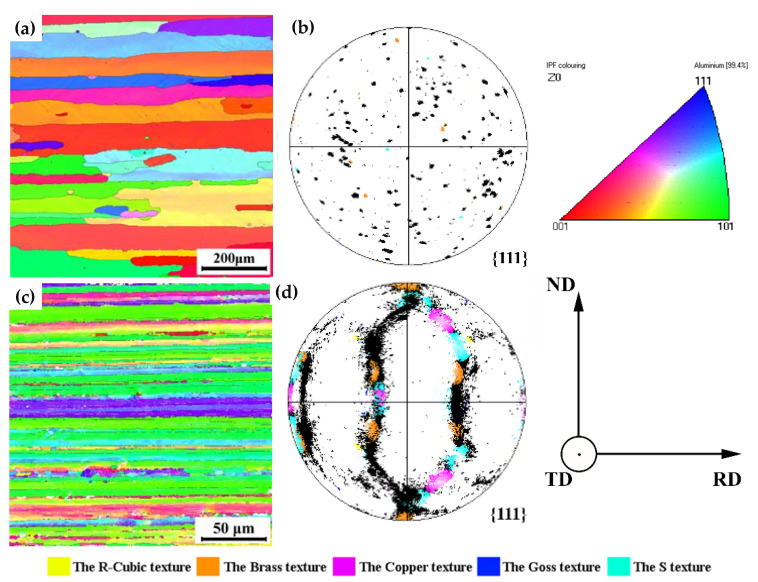
The orientation distribution maps and {111} pole figures of (**a**,**b**) AA6082 and (**c**,**d**) AA7204.

Figure 2 shows the orientation distribution maps of AA6082 and AA7204 alloys. The grains are colored according to their crystallographic orientation, as indicated by the color code. The volume fraction (VOF) of the grains, which nears green colors (corresponding to <110>||RD crystal direction), is high in the AA7204 sheet, indicating that the VOF of the {110}<uvw> components, such as the brass or goss components, is higher compared with AA6082 sheets [48]. In addition, the VOFs of the brass, copper, and S textures in AA7204 are 17.0%, 6.13%, and 10.8%, respectively.

**Figure 3:**
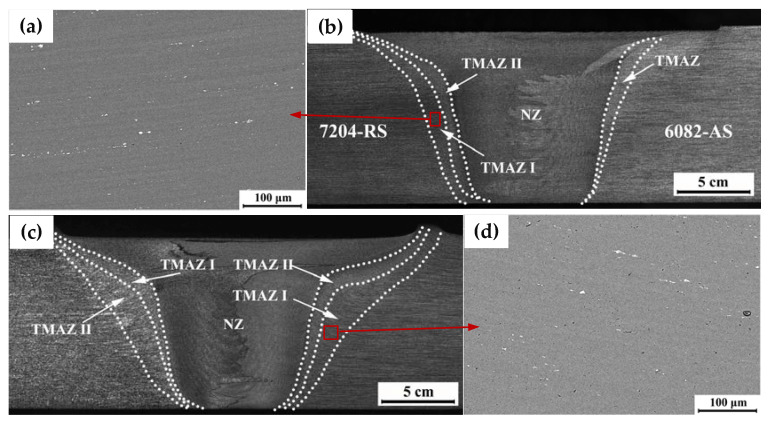
The macrostructure of (**a**,**b**) 7204-RS and (**c**,**d**) 7204-AS joints.

Macroscopic views of the 7204-RS and 7204-AS joints are shown in Figure 3b,c. Each welded joint can be divided into four zones: the NZ, TMAZ, HAZ, and BM. There is a great difference in the microstructure of the NZ and TMAZ between the 7204-RS and 7204-AS joints. Due to the different placement positions of the BM, “onion rings” with different morphologies are formed, and the TMAZ of the AA7204 side in the 7204-RS and 7204-AS joints can be divided into two parts. TMAZ I, which is near the HAZ, is under the action of shear deformation and friction heat of the stirring needle. The grains of the TMAZ I are twisted and show the characteristics of strips of torsion. TMAZ II, which is near the NZ, is in direct contact with the surface of the stirring needle during the welding process, resulting in severe shear deformation. The grains of the TMAZ II are fully broken and even smaller than the NZ. Therefore, a fine-grain belt is formed between the NZ and TMAZ II. As shown in Figure 3, the TMAZ I of the AA7204 side in the 7204-AS joint is wider than the 7204-RS joint, which indicates that the BM has undergone different degrees of deformation and material flow in TMAZ I. Furthermore, the SEM figures of 7204-RS-TMAZ I and 7204-AS-TMAZ I are shown in Figure 3a,d. Compared to 7204-AS-TMAZ I, the shear effect of stirring on 7204-RS-TMAZ I is weaker, and the bending degree of the second phase is smaller.

**Figure 4:**
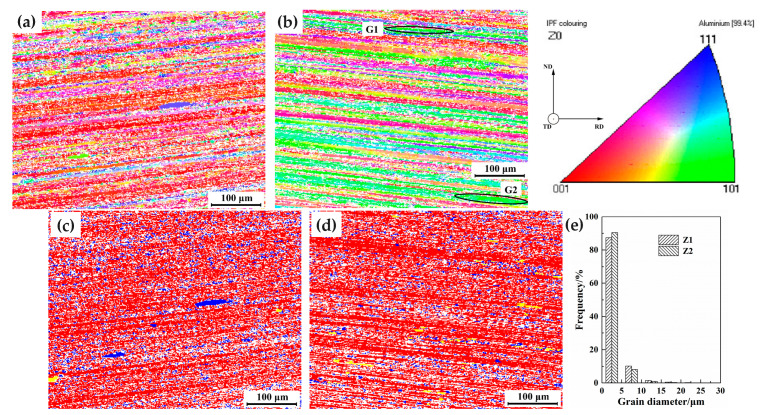
The (**a**,**b**) orientation distribution, (**c**,**d**) recrystallization distribution, and (**e**) grain size distribution maps of 7204-RS-TMAZ I and 7204-AZ-TMAZ I.

The red boxes in Figure 3a,b are labeled 7204-RS-TMAZ I and 7204-AZ-TMAZ I, and the orientation distribution maps of 7204-RS-TMAZ I and 7204-AZ-TMAZ I are shown in Figure 4. The structure of 7204-RS-TMAZ I and 7204-AZ-TMAZ I has obvious bending and maintained curved fibrous tissue. Because the positions of 7204-RS-TMAZ I and 7204-AZ-TMAZ I are the same distance as the welding area and the bending degrees are similar, the VOFs of deformed grains in 7204-RS-TMAZ I and 7204-AZ-TMAZ I are 88.23% and 90.95%, respectively, and the VOFs of recrystallized grains in 7204-RS-TMAZ I and 7204-AZ-TMAZ I are 11.62% and 7.99%, respectively. Both the deformed grains and recrystallized grains are evenly distributed. Furthermore, the average grain sizes of 7204-RS-TMAZ I and 7204-AZ-TMAZ I are 3.20 μm and 2.86 μm, respectively, and are mostly distributed between 0 and 5 μm. Therefore, the recrystallization and grain size of 7204-RS-TMAZ I and 7204-AZ-TMAZ I have no obvious difference. While the grain orientations of 7204-RS-TMAZ I and 7204-AZ-TMAZ I are quite different. The VOFs of the R-cubic ({001}<110>), brass copper, goss, and S texture in 7204-RS-TMAZ I are 0.84%, 0.30%, 0.71%, 0.01%, and 3.09%, respectively, and the VOFs of the R-cubic, brass copper, goss and S texture in 7204-AZ-TMAZ I are 0.83%, 5.17%, 1.47%, 0.02%, and 8.50%, respectively.

**Figure 5:**
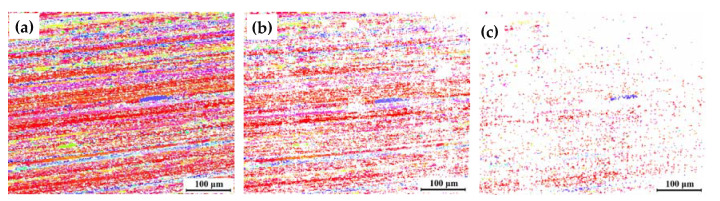
Orientation distribution maps of 7204-RS-TMAZ I after corrosion for (**a**) 0 h, (**b**) 1 h, and (**c**) 2 h.

Figure 5 shows the orientation distribution maps of 7204-RS-TMAZ I after corrosion for 0 h, 1 h, and 2 h. It should be emphasized that the white area in Figure 5 is the preferentially severely corroded region that could not be detected by the EBSD system. The VOF of the remaining structure decreases from 65% to 32.6% after corrosion for 1 h. In this process, local corrosion occurs and develops uniformly, and the corrosion behaviors do not correlate with grain orientation. Then, the VOF of the remaining structure decreases from 32.6% to 5.8% after corrosion from 1 h to 2 h. In this process, almost all structures are corroded, and the remaining microstructure is sporadically distributed in the matrix, which indicates that the structure in 7204-RS-TMAZ I has homogeneous corrosion resistance. The serious corrosion behavior indicates that TMAZ I of the AA7204 side in the 7204-RS joint has poor corrosion performance.

**Figure 6:**
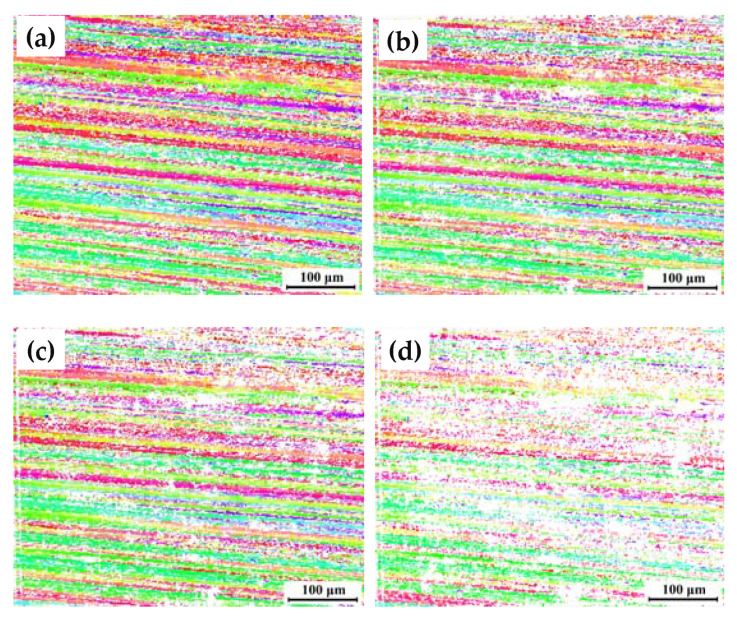
Orientation distribution maps of 7204-AZ-TMAZ I after corrosion for (**a**) 0 h, (**b**) 1 h, (**c**) 2 h, and (**d**) 4 h.

Figure 6 shows the orientation distribution maps of 7204-AZ-TMAZ I after corrosion for 0 h, 1 h, 2 h, and 4 h. The VOF of the remaining structure decreases from 66.9% to 59.3% after corrosion for 1 h, and in this process, local corrosion occurs and develops, while the degree of corrosion is very low. The VOF of the remaining structure decreases from 59.3% to 52.7% after corrosion from 1 h to 2 h. The local corrosion develops further and shows a correlation between corrosion behavior and grain orientation. As shown in Figure 6c, the structure with a grain orientation close to red is corroded rapidly, while the structure with a grain orientation close to green is retained, which shows good corrosion resistance. The VOF of the remaining structure decreases from 52.7% to 29.0% after corrosion from 2 h to 4 h. The local corrosion develops further, and the corrosion area overlaps. Furthermore, a correlation between local corrosion and grain orientation still exists. The corrosion phenomenon indicates that the TMAZ I of the AA7204 side in the 7204-AS joint has excellent corrosion resistance.

## 4. Discussion

**Figure 7:**
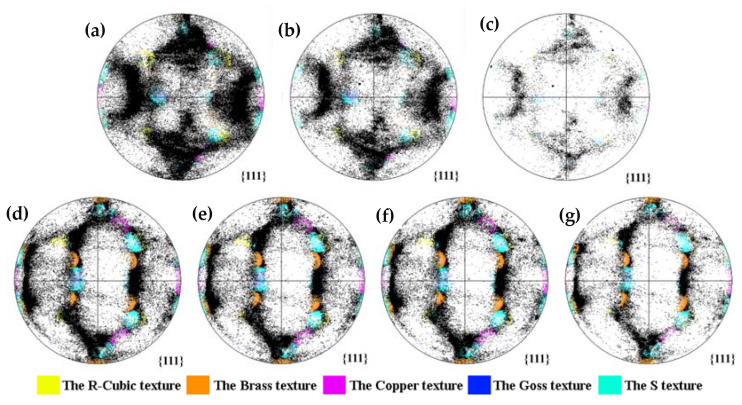
{111} pole figures of 7204-RS-TMAZ I after corrosion for (**a**) 0 h, (**b**) 1 h, (**c**) 2 h, and 7204-AZ-TMAZ I after corrosion for (**d**) 0 h, (**e**) 1 h, (**f**) 2 h, (**g**) 4 h.

The {111} pole figures with increasing corrosion time of 7204-RS-TMAZ I and 7204-AZ-TMAZ I are shown in Figure 7. There is no obvious texture in 7204-RS-TMAZ I, and almost all types of texture and matrix disappear after the quasi in situ corrosion test, which indicates that the weak texture in 7204-RS-TMAZ I does not show good corrosion resistance in the corrosion process and the corrosion of 7204-RS-TMAZ is poor. There is a strong texture in 7204-AZ-TMAZ I. Except for a small part of the dispersed distribution, most of the grain orientations are concentrated in specific areas of the {111} polar figures, excluding the identified texture. Most grains near the strong texture, especially the brass and S textures, are retained after corrosion for 4 h, while the grains with random grain orientation are almost corroded. Therefore, the strong texture, especially the brass and S textures, shows excellent corrosion resistance during the corrosion process, and the corrosion resistance of 7204-AZ-TMAZ is excellent.

**Figure 8:**
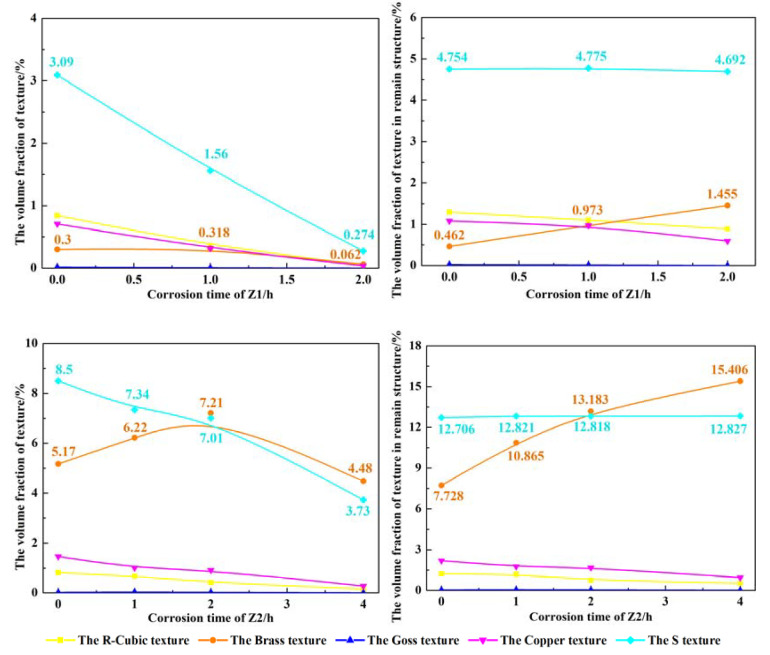
The VOF of the remaining R-cubic, brass, goss, copper, and S textures during the corrosion test.

To further analyze the corrosion resistance of different types of textures, the VOF changes of the R-cubic, brass, goss, copper, and S textures of 7204-RS-TMAZ I and 7204-AZ-TMAZ I with increasing corrosion time are shown in Table 2 and Table 3 and Figure 8. For 7204-RS-TMAZ I, the VOF in the total area of the R-cubic texture reduces from 0.84% to 0.05%, the brass texture reduces from 0.30% to 0.06%, the goss texture reduces from 0.01% to 0%, the copper texture reduces from 0.70% to 0.03%, and the S texture reduces from 3.09% to 0.27% after corrosion for 2 h. The VOF in the remaining structure of the R-cubic texture reduces from 1.29% to 0.88%, the brass texture increases from 0.46% to 1.45%, the goss texture reduces from 0.02% to 0%, the copper texture reduces from 1.08% to 0.59%, and the S texture reduces from 4.75% to 4.69% after corrosion for 2 h. For 7204-AZ-TMAZ I, the VOF in the total area of the R-cubic texture reduces from 0.82% to 0.16%, the goss texture reduces from 0.02% to 0%, the copper texture reduces from 1.47 to 0.27, and the S texture reduces from 8.50% to 3.73% after corrosion for 4 h, while the VOF in the total area of the brass texture increases from 5.17% to 7.21% after corrosion for 2 h and reduces from 7.21% to 4.48% after corrosion from 2 h to 4 h. The VOF in the remaining structure of the R-cubic texture reduces from 1.23% to 0.53%, the brass texture increases from 7.72% to 15.41%, the goss texture reduces from 0.02% to 0.01%, the copper texture reduces from 2.19% to 0.96%, and the S texture increases from 12.71% to 12.83% after corrosion for 4 h.

Therefore, the VOF of all types of textures in the total area and the VOF of the R-cubic and copper textures in the remaining structure are significantly reduced with increasing corrosion time, while the VOF of the S texture in the remaining structure remains unchanged and the brass texture increases significantly. This indicates that the grains defined as the R-cubic and copper textures have poor corrosion resistance, the S texture has the same corrosion resistance as the matrix, and grains defined as the brass texture have excellent corrosion resistance. Furthermore, a similar correlation between texture and corrosion resistance is observed in 7204-AZ-TMAZ I. However, it is contrary to common sense that the VOF of the brass texture in the total area of 7204-AZ-TMAZ I increases from 5.17% to 7.21% during corrosion for 2 h; this may be the reason for the variable corrosion rate of 7204-AZ-TMAZ I after corrosion for 4 h.

**Figure 9:**
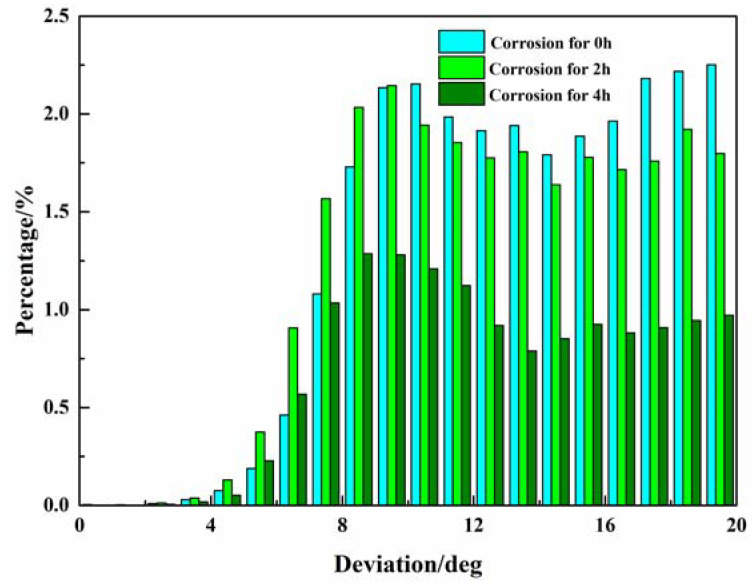
VOF distribution diagram of the ODA between the grain orientation and {110}<211> within 20° after corrosion for 0, 2, and 4 h.

In the previous statement, the structure with an ODA between grain orientation and {110}<211> within 10° is defined as the brass texture, and the grain with an ODA between grain orientation and {110}<211> from 10° to 20° seems beneficial to the increase in the brass texture during corrosion. Figure 9 shows the VOF distribution diagram of the ODA between the grain orientation and {110} <211> within 20° after corrosion for 0 h, 2 h, and 4 h. The VOF of the structure with ODAs within 20° changes from 26.0% to 25.2% after corrosion for 2 h. The VOF of the grain structure with all the ODA within 10° increases, and the ODA from 6° to 9° shows the greatest increase, while the VOF of the grain structure with all the ODA from 10° to 20° decreases, and the ODA from 16° to 20° shows the greatest reduction. The grain structure with a high ODA between the grain orientation and {110}<211> changed to approaching {110}<211> due to corrosion, which indicates that the grain orientation is closer to {110}<211> and the corrosion resistance is better.

The VOF of the grain ODA within 20 ° changes from 25.2% to 14.0% after corrosion from 2 h to 4 h, and the reduction in the VOF of the grain structure reaches approximately 50% with the ODA from 0° to 5° and 12° to 20°. The reduction in the VOF of the grain structure is approximately 40% with the ODA from 5° to 12° after corrosion from 2 h to 4 h. That is, the VOF of the grain structure with all ODAs from 0° to 20° decreases, and the heterogeneity of the corrosion degree also indicates that the grains with different grain ODAs with {110}<211> have different corrosion resistances.

To further analyze the transformation behavior of the brass texture in the corrosion process, the grains shown in Figure 4, in which most structure orientations are close to {110}<211>, are selected for grain orientation change analysis. It must be noted that the closer the grain orientation of the red grain structure is to {110}<211>, the greater the grain orientation of the blue grain structure deviates from {110}<211>.

**Figure 10:**
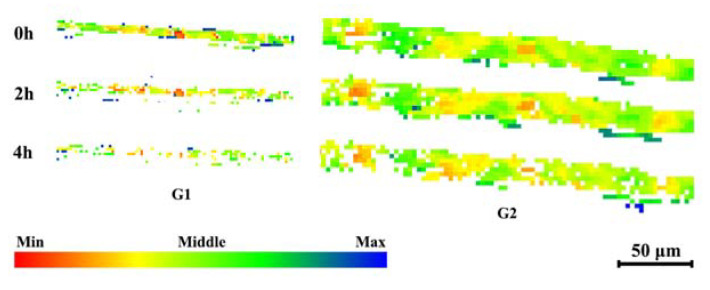
The grain orientation change in the corrosion test.

Figure 10 shows a slight difference in the orientation within the grains. For G1, the microstructure with grain orientation closer to {110}<211> shows better corrosion resistance during corrosion for 2 h, while with the corrosion from 2 h to 4 h, severe corrosion occurs in grain G1. For G2, the structure with grain orientation closer to {110}<211> is retained because of better corrosion resistance, while the grain structure with grain orientation is farther away from the standard orientation, and the brass texture is corroded slightly. That is, the corrosion inside the grains is uneven, and the grains are not completely corroded, which can still be detected in subsequent EBSD detection. Therefore, the grain orientation after corrosion changes slightly from before corrosion, and the experimental data show that the grain orientation of G2 is turning toward {110}<211>, which greatly improves the corrosion resistance of the TMAZ I of the AA7204 side in the 7204-AS joint. It indicates that corrosion, like deformation, is carried out alongside the {110} plane for the structure with grain orientation near {110}<112>.

With increasing corrosion time, the grain structure with less poor corrosion performance around the brass texture is corroded. The contact area between the grain structure defined as the brass texture and the corrosion solution increases the corrosion rate of the brass texture, causing the VOF of the brass texture area to decrease after corrosion from 2 to 4 h.

From what is discussed above, it can be seen that the grains, which are distributed in different locations, show different corrosion behaviors. To analyze the cause of this difference, the actual evolution process of the R-cubic, brass, goss, copper, and S textures during the corrosion process of the 7204-AZ-TMAZ I after corrosion for 0, 1, 2, and 4 h is obtained.

**Figure 11:**
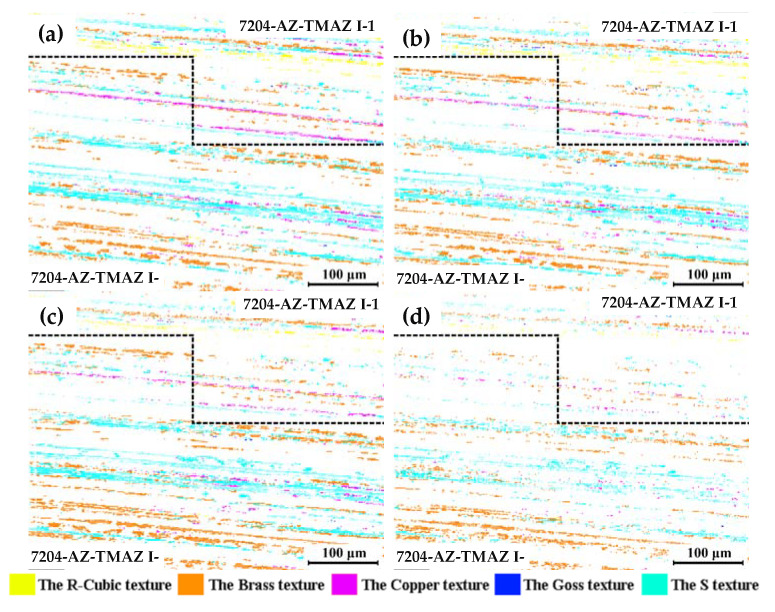
Texture evolution of the R-cubic, brass, goss, copper, and S textures after corrosion for (**a**) 0 h, (**b**) 1 h, (**c**) 2 h, and (**d**) 4 h at 7204-AZ-TMAZ I.

Figure 11a can be divided into 7204-AZ-TMAZ I-1 and 7204-AZ-TMAZ I-2 according to the difference in the corrosion behavior of the brass texture. A large number of the R-cubic and copper textures are distributed around the brass texture in 7204-AZ-TMAZ I-1, and the corrosion resistance of the R-cubic and copper textures is worse than the matrix, which worsens the corrosion performance of 7204-AZ-TMAZ I-1. After the corrosion of 1 h, the VOF of the partial brass texture increases, and the VOF of the other brass textures decreases, while the VOF of the brass texture decreases after corrosion for 2 h and 4 h. In 7204-AZ-TMAZ I-2, there are a large number of S textures and a small amount of sporadic copper texture distributed around the brass texture. The brass texture has better corrosion resistance than the matrix, and the corrosion resistance of the S texture is equivalent to the matrix, so 7204-AZ-TMAZ I-2 has excellent corrosion resistance and a slow corrosion rate. Therefore, the VOF of the brass texture increases after corrosion for 2 h, and a local decrease in the brass texture occurs after corrosion from 2 to 4 h. And a small part of the brass texture still increases further.

The VOF of the brass texture after corrosion for 0, 1, 2, and 4 h is 1.15%, 1.09%, 0.92%, and 0.48% of the 7204-AZ-TMAZ I-1, respectively, and the VOF of the brass texture after corrosion for 0, 1, 2, and 4 h is 4.02%, 5.13%, 6.30%, and 3.99% of zone 22, respectively. That is, the local decrease in the brass texture in 7204-AZ-TMAZ I-1 is less than the local increase in the brass texture in 7204-AZ-TMAZ I-1 and 7204-AZ-TMAZ I after corrosion for 2 h. And the local increase in the brass texture in 7204-AZ-TMAZ I-2 is much less than the decrease in the brass texture in 7204-AZ-TMAZ I-1 and 7204-AZ-TMAZ I-2 after corrosion from 2 h to 4 h. Therefore, the VOF of the brass texture increases from 5.17% to 7.21% after corrosion for 2 h and then decreases from 7.21% to 4.48% after corrosion from 2 h to 4 h, as shown in Figure 8c. However, compared with the matrix, the corrosion rate of the brass texture is relatively slow, and the brass texture still shows good corrosion resistance at corrosion from 2 h to 4 h.

Therefore, the corrosion resistance of TMAZ II near AA7204 is not only influenced by the chemical effect but also affected by different texture configurations on the AA7204 side of the FSW joints. By controlling the texture configuration, the corrosion behavior of the texture would be changed, and the application of FSW joints could be improved.

## 5. Conclusions

The results obtained from this study of AA6082/AA7204 dissimilar welded joints produced by FSW are summarized as follows:

(1) The structure with grain orientation close to the brass texture ({110}<112>) has excellent corrosion resistance;
(2) The corrosion is carried out alongside the {110} plane for the brass texture around the S texture;
(3) The corrosion resistance of 7204-AZ-TMAZ I will be improved by optimizing the texture configuration of the brass texture 17 times and the S texture 2.75 times more than 7204-RS-TMAZ I.

## Figures and Tables

**Table 1:** Chemical compositions of AA6082-T6 and AA7204-T4 alloys (wt%).

BM	Si	Fe	Cu	Mg	Zn	Mn	Cr	Zr	Ti	V	Al
AA6082-T6	0.917	0.216	0.011	0.766	0.024	0.520	0.005	/	0.026	/	Bal
AA7204-T4	0.044	0.08	0.14	1.34	4.82	0.36	0.17	0.13	0.057	/	Bal

**Table 2:** The volume fraction of the texture during the corrosion test/%.

Sample	Corrosion Time/h	R-Cubic Texture	Brass Texture	Goss Texture	Copper Texture	S Texture
7204-RS	0	0.842	0.300	0.015	0.706	3.090
	1	0.361	0.318	0.005	0.318	1.560
	2	0.052	0.062	0.000	0.035	0.274
7204-AS	0	0.825	5.170	0.019	1.470	8.500
	1	0.683	6.220	0.033	1.010	7.340
	2	0.418	7.210	0.020	0.921	7.010
	4	0.155	4.480	0.003	0.278	3.730

**Table 3:** The volume fraction of the texture in the remaining structure during the corrosion test/%.

Sample	Corrosion Time/h	R-Cubic Texture	Brass Texture	Goss Texture	Copper Texture	S Texture
7204-RS	0	1.295	0.462	0.022	1.080	4.754
	1	1.105	0.973	0.013	0.973	4.775
	2	0.889	1.455	0.000	0.592	4.692
7204-AS	0	1.233	7.728	0.029	2.197	12.706
	1	1.193	10.865	0.057	1.764	12.821
	2	0.764	13.183	0.037	1.684	12.818
	4	0.533	15.406	0.012	0.956	12.827

## Data Availability

Not applicable.
